# Paternal High Fat Diet in Rats Leads to Renal Accumulation of Lipid and Tubular Changes in Adult Offspring

**DOI:** 10.3390/nu8090521

**Published:** 2016-08-23

**Authors:** Sabiha S. Chowdhury, Virginie Lecomte, Jonathan H. Erlich, Christopher A. Maloney, Margaret J. Morris

**Affiliations:** 1 School of Medical Sciences, University of New South Wales, Sydney 2052, NSW, Australia; sabiha.chowdhury@unsw.edu.au (S.S.C.); v.lecomte@unsw.edu.au (V.L.); c.maloney@unsw.edu.au (C.A.M.); 2 Prince of Wales Clinical School, University of New South Wales, Sydney 2052, NSW, Australia; j.erlich@unsw.edu.au; 3 Department of Nephrology, Prince of Wales Hospital, Randwick 2031, NSW, Australia

**Keywords:** paternal diet, high fat diet, obesity, programming, kidney, triglyceride

## Abstract

Along with diabetes and obesity, chronic kidney disease (CKD) is increasing across the globe. Although some data support an effect of maternal obesity on offspring kidney, the impact of paternal obesity is unknown; thus, we have studied the effect of paternal obesity prior to conception. Male Sprague Dawley rats were fed chow diet or high fat diet (HFD) for 13–14 weeks before mating with chow-fed females. Male offspring were weaned onto chow and killed at 27 weeks for renal gene expression and histology. Fathers on HFD were 30% heavier than Controls at mating. At 27 weeks of age offspring of obese fathers weighed 10% less; kidney triglyceride content was significantly increased (5.35 ± 0.84 vs. 2.99 ± 0.47 μg/mg, *p* < 0.05, *n* = 8 litters per group. Histological analysis of the kidney demonstrated signs of tubule damage, with significantly greater loss of brush border, and increased cell sloughing in offspring of obese compared to Control fathers. Acat1, involved in entry of fatty acid for beta-oxidation, was significantly upregulated, possibly to counteract increased triglyceride storage. However other genes involved in lipid metabolism, inflammation and kidney injury showed no changes. Paternal obesity was associated with renal triglyceride accumulation and histological changes in tubules, suggesting a mild renal insult in offspring, who may be at risk of developing CKD.

## 1. Introduction

The prevalence of obesity is increasing around the world across all ages. In recent years, childhood obesity has been of particular concern as it is rising at a faster rate in both developed and developing countries, and has been linked to the emergence of metabolic disease in early life [1]. In parallel, chronic kidney disease (CKD) is also a growing public health concern and is recognized as a common condition that elevates the risk of cardiovascular disease, kidney failure and other complications [2]. Of even more concern, over the last three decades a significant increase of CKD has been reported in the paediatric population [3].

It has been well established using animal models that parental metabolic status at conception can influence offspring metabolic health outcomes. Effects of maternal diet on offspring have been extensively studied with data showing that maternal obesity can program offspring health, making them more prone to obesity, diabetes and hypertension [4,5]. It is becoming clear that paternal diet also has consequences for offspring health. Several labs, including our own, have demonstrated that paternal obesity can also alter the metabolic status as well as reproductive capacities of offspring [6,7,8]. In terms of renal programming, extensive work in rodents has shown that maternal undernutrition and caloric restriction can affect offspring nephrogenesis and kidney structure, contributing to adverse functional outcomes [9].

While there is evidence for the impact of maternal factors on offspring kidney across a range of species [10], there has been little investigation to date of the issue of whether paternal dietary factors can affect offspring kidney. Vik et al. reported similar father-offspring and mother-offspring associations across cardiovascular risk factors [11]. A cross sectional study of 580 Chinese children revealed that paternal BMI was associated with cardiovascular risk factors [12]. Others have shown that paternal BMI is correlated with offspring body fat, BMI and birth parameters as well as increased cardiometabolic risk in a sex dependent manner [13,14]. 

Given the increasing rates of obesity in the population, and the potential role of paternal obesity in programming offspring health outcomes [15], the aim of the present study was to investigate the effects of paternal obesity on offspring kidney. This came out of a striking observation of gross changes in the appearance of kidneys from adult offspring of obese compared to lean fathers; their kidneys showed signs of lipid accumulation. Our aim was to test for morphological changes in offspring kidneys, as well as gene expression of the markers of kidney injury Kim 1 and Ngal [16,17]. We also examined the triglyceride content of the kidney and expression of genes involved in triglyceride accumulation including lipogenic genes and inflammatory markers. 

## 2. Materials and Methods 

### 2.1. Animals

Three week old male Sprague Dawley (SD) rats were purchased from the Animal Research Centre (ARC, Perth, Australia) and divided into two groups of equal starting body weight. Control rats were fed control chow diet (11 kJ/g, 12% fat, 21% protein, 65% carbohydrate as percent energy; Gordon’s Stockfeeds, Yanderra, NSW, Australia) whilst the high-fat diet (HFD) group was provided the control chow, and two commercial high fat pelleted diets, SF03-020 (20 kJ/g, 43% fat, 17% protein, 40% carbohydrate) and SF01-025 (18.3 kJ/g, 44% fat, 17% protein, 39% carbohydrate; Specialty feeds, Glen Forest, WA, Australia). The rats were housed under a 12 h light: dark cycle. After 13–14 weeks of diet, male Control and HFD rats were mated with chow diet fed female SD rats. Mating was conducted, during daylight hours, and rats were returned to their home cages overnight, in order to maintain females on chow diet and males on their respective diets (HFD or chow) during the night time. Females remained on chow diet throughout gestation and lactation. At weaning on post-natal day 21, male offspring were given chow diet, and were housed four per cage up to 8–10 weeks of age, then two per cage up to 27 weeks of age. Male offspring were culled at 27 weeks of age after anaesthesia induced by i.p. injection of 100 mg ketamine and 15 mg xylazine/kg body weight, followed by decapitation. Blood was collected by cardiac puncture, before the decapitation, into heparin coated tubes; plasma samples were obtained after centrifugation and stored at −20 °C. The left kidney was snap frozen in liquid nitrogen at collection and stored at −80 °C. The right kidney was fixed in 10% formalin solution for 24 h, washed and then stored in 70% ethanol solution for subsequent histopathology analysis. Retroperitoneal (Rp) and epididymal (Epi) white adipose tissue (WAT) were weighed. Animal work was approved by the Animal Care and Ethics Committee of UNSW Australia (ACEC #11/82B).

### 2.2. Kidney Triglyceride Measurement

To measure triglyceride content, kidney was ground and homogenized using a chloroform-methanol mixture (2:1) in a Precellys 24 homogenizer (Sapphire Bioscience, Sydney, NSW, Australia). The homogenate was transferred to glass tubes, vortexed and placed on a roller mixer overnight. Two ml of 0.6% NaCl was added and centrifuged for 10 min at room temperature (1000× *g*). The entire organic phase was collected into another glass tube and evaporated under nitrogen gas in a heating block at 40 °C for 30 min. The dried extract was dissolved in absolute ethanol. Triglyceride content analysis was done by colorimetric assay using commercially available triglyceride reagent (Roche Diagnostic, Castle Hill, Australia) and standard (Sigma, St. Louis, MO, USA). Concentration was calculated by measuring absorbance at 490 nm using a BioRad iMark plate reader (BioRad, Sydney, NSW, Australia). 

### 2.3. Plasma Lipid, Electrolytes, Creatinine, Albumin and Cystatin C Measurements

Plasma NEFA (non-esterified fatty acids) concentrations were measured using a NEFA C kit (Wako Pure Chemicals Industries, Osaka, Japan). Plasma triglyceride concentrations were measured by the method described above for kidney triglyceride. Plasma creatinine and albumin concentrations were measured using a Konelab TM 20XT clinical chemistry analyser (Thermo Scientific, Waltham, MA, USA). Plasma electrolytes were measured using a GEM Premier 3500 (Instrumentation laboratory, Bedford, MA, USA). Plasma cystatin C was measured using a cystatin C duoset (catalogue number DY1238, R & D systems, Minneapolis, MN, USA).

### 2.4. Quantitative Polymerase Chain Reaction (q-PCR)

RNA was extracted using Tri-reagent (Sigma) and treated with DNAse (Ambion) to remove any contaminating genomic DNA and stored at −80 °C. RNA concentration was measured using a Biospec-nano spectrophotometer (Shimadzu, Sydney, NSW, Australia). Two micrograms of RNA were reverse transcribed to cDNA using High capacity reverse transcription kit (Applied Biosystems from Life Technology, Foster City, CA, USA) and stored in −30 °C. Quantitative polymerase chain reaction q-PCR was performed using pre-optimized TaqMan probe/primers (Applied Biosystems from Life Technology, Foster City, CA, USA) including Cd36 molecule (Cd36), carnitine palmitoyltransferase 1A (Cpt1α), fatty acid synthase (Fasn), hydroxyacyl-CoA dehydrogenase (Hadh), peroxisome proliferator-activated receptor gamma, coactivator 1 alpha (Pgc1α), sterol regulatory element binding protein 1 and 2 (Srebp1, Srebp 2), interleukin 6 (IL-6), monocyte chemoattractant protein-1 (Mcp1), transforming growth factor beta 1 (Tgf β1), tumor necrosis factor alpha (Tnf-α), peroxisome proliferator-activated receptor gamma (Pparg), acetyl-CoA acetyltransferase 1 (Acat1), acetyl-CoA Carboxylase Alpha (Acaca), low density lipoprotein receptor (Ldlr), lipoprotein lipase (Lpl), kidney injury molecule 1 (Kim 1), neutrophil gelatinase-associated lipocalin (Ngal) and were normalized to the geometric mean of β-actin and Hprt 1 (housekeeping genes identified in preliminary experiments) using a pooled sample as calibrator. Five housekeepers were assessed and the stability of each was analysed using Normfinder software [18]. β-actin and Hprt 1 showed the highest stability value and Ct values were consistent across treatment groups. Analysis was performed using the ∆∆CT method [19]. q-PCR was performed using QuantStudio 12K Flex (Life technologies, Waltham, MA, USA) instrument and software.

### 2.5. Histology 

Histomorphological analysis was performed on periodic acid Schiff (PAS) stained five micron thick paraffin embedded tissue sections. Tubular damage was assessed by an operator blinded to the treatment groups using a scoring system looking at the proportion of damaged tubules. Thirty non-overlapping fields from each kidney were captured using an Olympus BX51microscope, 20X objective, and Neurolucida Explorer software (MBF Bioscience, Williston, VT, USA). The proportion of tubules displaying missing or ruptured brush border, presence of debris and apoptotic cells (assessed by chromatin condensation), were evaluated using a modification of the method of Melnikov et al. [20]. 

### 2.6. Statistical Analysis

Statistical analyses were performed using unpaired Student’s *t*-test after checking for normality. Data are presented as mean ± SEM. For offspring, body weight and post-mortem data represent the average of one to two pups per litter (*n* = 8 litters per group). Histological data represent five offspring from five different fathers in each group and mRNA data represent the average of eight offspring, each derived from a separate father. Differences were considered significant at *p* < 0.05.

## 3. Results

### 3.1. Effect of HFD on Fathers

Male HFD fed fathers consumed more energy than Control rats (cumulative energy intake 59,326 ± 3115 kJ and 43,946 ± 3820 kJ respectively, *p* < 0.0001) and at the time of mating were 30% heavier (mean body weight 710.7 ± 25.4 g and 530.0 ± 23.1 g respectively, *n* = 8 per group, *p* < 0.001). WAT mass (41.3 ± 4.2 g and 15.3 ± 1.4 g, *n* = 8 and *n* = 7 per group, *p* < 0.001) and liver mass (20.2 ± 1.1 g and 15.1 ± 1.3 g, *n* = 8 and *n* = 7 per group, *p* < 0.01) were significantly increased in HFD versus Control fathers. 

### 3.2. Offspring Body Weight, Tissue Mass and Kidney Triglyceride at Week 27

At 27 weeks of age, male offspring of obese fathers had significantly lower body weights (10% lower) compared to offspring from lean fathers (*p* < 0.01, Table 1). Kidney weight showed no significant differences between the groups. Liver and fat mass were both significantly reduced by 15% and 24% respectively, in the offspring of obese fathers (*p* < 0.05, Table 1). However, these effects were not sustained following correction for body weight (data not shown). Paternal obesity was associated with a significant 84% increase in kidney triglyceride concentrations compared to Control offspring (*p* < 0.05, Table 1), whereas there was no effect on plasma triglyceride and NEFA concentrations.

### 3.3. Plasma Creatinine, Albumin, Electrolyte and Cystatin C Concentrations

Plasma measures of kidney function (creatinine, albumin, electrolytes and cystatin C concentrations) were not altered between offspring from Control or HFD fathers (Table 2). 

### 3.4. Renal Gene Expression

Changes in renal lipid synthesis, uptake and β-oxidation, along with inflammation were assessed by determining the expression of genes. Among the genes involved in lipid metabolism (Cd36, Ldlr, Srebp1, Srebp2, Acaca, Fasn, Lpl) examined, Acat1 was significantly upregulated in offspring of obese fathers (Table 3, *p* < 0.05), with no significant changes in the expression of the other genes. We measured expression of genes involved in β-oxidation (Hadh, Cpt1a, Pgc1α, Pparg), inflammatory markers including Tnf-α, IL-6, Mcp1a, and the pro-fibrotic gene (Tgf β1), but no significant differences were observed between offspring of lean and obese fathers (Table 3).

### 3.5. Kidney histology

**Figure 1:**
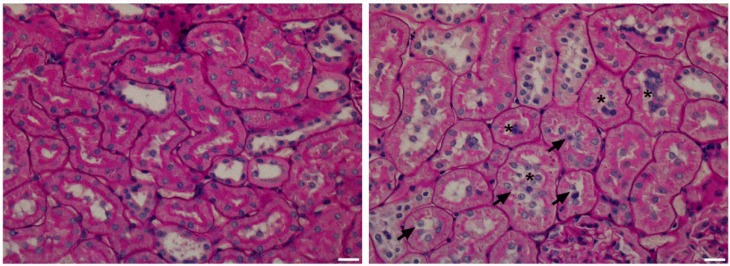
Histological images of representative periodic acid Schiff (PAS) stained kidney sections (400×, scale bar represents 20 μm). PAS stained kidney sections of a rat from Control (**left**) and HFD (**right**) group showing greater evidence of missing or ruptured brush border (arrows) and cell sloughing inside the lumen (asterisk) in offspring of HFD fed fathers compared to Control.

Histological analysis of PAS stained sections of five kidneys from five different fathers per group revealed evidence of increased tubular damage in the kidneys of offspring born to obese fathers. We observed a significant increase of cell sloughing inside the tubular lumen and ruptured brush border in tubules of offspring of HFD compared to Control fathers (Table 4, Figure 1). Kidney triglyceride content was significantly correlated with the degree of cell sloughing observed (*r*^2^ = 0.8145, *p* < 0.001). There was no difference in apoptotic cells and debris. No sclerosis or marked glomerular abnormalities were seen in any of the specimens.

Having noted changes in the brush border of tubular epithelial cells and an apparent increase in cell sloughing in the kidneys from offspring of obese fathers, we next measured the expression of two markers of acute kidney injury, Ngal and Kim1, neither of which differed between groups (Table 3).

## 4. Discussion

The increasing prevalence of obesity is now contributing to the burden of kidney disease [21]. We show for the first time that kidneys from rat offspring born to obese fathers had subclinical renal injury with signs of tubular damage and increased triglyceride accumulation compared to those from lean fathers. The impact of maternal overnutrition on offspring kidney has gained attention more recently but there has been limited investigation on the effects of HFD consumption by fathers on the renal function of their offspring. A human epidemiological study examining cystatin C, a biomarker of renal function, showed that higher levels of cystatin C, and hence presumed reduced renal function in either parent, resulted in higher cystatin C in the offspring [22]. This is one of the only studies to investigate the association between paternal factors and their offspring kidney function. 

In this study, we describe early signs of offspring renal injury in a model of paternal obesity. The male offspring of obese fathers had evidence of tubular injury with loss of brush borders and sloughing of cells inside the lumen. These are common findings in animal studies of acute and chronic kidney injury [23] as well as biopsy of humans with kidney disease [24]. It has been shown that renal disease has been associated with lipotoxicity in the kidney, and triglyceride accumulation is an early sign [25]. Interestingly with tubular damage we observed increased renal triglyceride content but we did not observe overt hypertriglyceridemia in these offspring of obese fathers. From our current data we cannot determine the specific renal site or sites of triglyceride accumulation; renal cortex and proximal tubular accumulation of lipid have been reported in a number of circumstances [26]. Johnson et al. reported that it occurs preferentially in proximal tubule of rat kidneys where there is an increased filtered load resulting from glomerular injury; this increase in delivered lipid load could result in lipid accumulation [27]. Lipid accumulation can cause lipotoxicity and energy stress, which may further contribute to CKD pathogenesis [28,29]. In our model, although we did not observe any signs of glomerular injury, we observed a significant positive correlation between triglyceride content in the kidney and signs of tubular injury.

Renal lipid accumulation may result from either defective parenchymal lipid processing or increased delivery [30] and contribute to progressive renal injury such as that observed in diabetic nephropathy [31]. As we found no changes in plasma triglyceride levels, our data tend to suggest a problem with renal lipid processing. To explore possible reasons underpinning the triglyceride accumulation in kidneys of offspring born to obese fathers, we examined genes involved in lipid transport, synthesis, and oxidation. We observed an upregulation of Acat1, which is involved in oxidation of lipid probably to counteract the increased triglyceride content. Although ACAT1 is the only gene showing a differential expression among those we measured, it might be a consequence of the phenotype and not a main programming event. It seems that the kidneys of offspring from obese fathers do not have a predisposition to lipid accumulation due to a ‘programmed’ change in expression of the genes we have measured. Further insults such as overnutrition with HFD may worsen the kidneys of such offspring, and this would be interesting to follow up in further studies.

A study by Jackson et al. found that maternal HFD can cause glomerulosclerosis and tubulointerstitial fibrosis in male offspring kidney [32] by activating pro-inflammatory pathways. At present, we cannot pinpoint a clear mechanism for the injuries described in our offspring. Generation of reactive oxygen species and release of pro-inflammatory and pro-fibrotic factors triggered by renal lipid accumulation [33] has been suggested as a mechanism leading to tubular damage. We observed accumulation of triglyceride along with tubular damage in kidneys from offspring of obese fathers; however, we did not see any changes in expression of inflammatory and kidney injury markers. Thus we may have observed an early sign of injury in our offspring.

Plasma creatinine is a relatively blunt marker of renal injury. Creatinine, the commonly used biomarker for glomerular filtration rate (GFR), often does not change until there is already substantial renal parenchymal injury making it a late marker of renal injury [34]. Moreover, eventual reduction of GFR without signs of CKD has been associated with an increased risk of adverse cardiovascular outcomes [35]. Thus a major risk factor for progressive kidney injury and loss of GFR is underlying subclinical CKD. Taking into account the type of tubular injury we observed and relative lack of tubular atrophy and interstitial fibrosis it was not surprising that there were no significant changes in creatinine or plasma electrolyte concentrations. Blood pressure was not assessed in these offspring, but given the early nature of the tubular injury observed in the kidney of offspring from obese fathers, it is unlikely that such a major parameter would be affected. The fact that there was no change in plasma creatinine or electrolyte concentrations supports this hypothesis. One limitation of the current study is that we were unable to access urine. Further investigation of urine and plasma biomarkers of renal injury such as urinary protein, may clarify the significance of the observed renal changes. However, gene expression of biomarkers of renal injury Kim1 and Ngal [16,17] were measured in the kidney of offspring from Control and obese fathers but no difference between groups were observed. This does not exclude more subtle cellular injury. Further study with urine collection will be required. Previous renal injury and scarring is an important risk for further renal injury and these offspring may be at increased risk for more severe injury with future challenges. 

Most studies investigating the effect of parental nutrition on kidney in the offspring have used maternal under nutrition during pregnancy, predominantly reporting changes in nephron number and glomerular volume [36]. In humans, low birth weight is associated with increased albuminuria [37], reduced GFR, and increased risk of CKD [38,39]. The link between HFD consumption or obesity in parents and risk to the offspring kidney has been examined more recently. A population based study reported that children of obese mothers have a 22% increased risk of developing CKD [40]. Work in rodents showed that maternal overnutrition (high fat/fructose diet) during pregnancy may increase susceptibility to renal injury in offspring [41]. Our report is the first to our knowledge suggesting a programming effect of paternal obesity on offspring kidney. There is evidence of sex specific effects of paternal exposures on offspring, and here we did not observe any noticeable kidney phenotype in the sisters of these males. There is also evidence that insults around the time of conception can have long lasting impacts on the kidney, and it is likely that these are mediated via epigenetic mechanisms including alterations in sperm micro-RNAs, histone acetylation and DNA methylation [13]. In this regard, our earlier work in the fathers used in this study revealed changes in overall DNA methylation in sperm, but these changes were not transmitted to offspring muscle, liver and sperm [42]. Here minimal gene changes were observed in the kidney, and further work is required to examine whether epigenetic mechanisms are at play.

## 5. Conclusions 

In conclusion, we found that paternal obesity can lead to increased triglyceride content in offspring kidneys with signs of tubular damage, such as cell sloughing, absence of brush border which may be indicative of early signs of kidney damage. This is the first report to our knowledge of an effect of paternal obesity, induced by HFD, on offspring kidney. While the effects are modest, it must be emphasized that the offspring were consuming a healthy chow diet, and were not obese themselves. Further work should explore whether this programming effect may worsen as offspring age, or if they are exposed to an unhealthy diet. Given the observations made in this study, offspring of obese fathers may have an altered response to renal injury contributing to the increased risk of kidney disease.

## Figures and Tables

**Table 1:** Offspring body weight, fat mass, organ mass, and kidney and plasma triglyceride concentrations.

Paternal Diet	Control (*n* = 8)	HFD (*n* = 8)
Body weight (g)	567.7 ± 12.1	504.1 ± 11.3 **
Fat mass (g)	14.56 ± 1.08	10.94 ± 1.14 *
Liver mass (g)	16.23 ± 0.75	13.71 ± 0.65 *
Kidney mass (g)	1.55 ± 0.09	1.55 ± 0.05
Kidney triglyceride (μg/mg)	2.99 ± 0.47	5.35 ± 0.84 *
Plasma triglyceride (mmol/L)	0.94 ± 0.13	0.90 ± 0.07
Plasma NEFA (mmol/L)	1.28 ± 0.22	1.18 ± 0.10

Body weight, organ mass, kidney and plasma triglyceride concentrations in male offspring of Control and HFD fed fathers at 27 weeks of age. Fat mass represents the sum of Rp WAT and Epi WAT mass. Data are presented as mean ± SEM of the average of one to two offspring from eight fathers in each diet group. Statistical analyses were performed using Student’s t-test (* *p* < 0.05, ** *p* < 0.01 compared to Control).

**Table 2:** Plasma creatinine, albumin, cystatin C and electrolyte concentrations.

Paternal Diet	Control (*n* = 8)	HFD (*n* = 8)
Creatinine (μmol/L)	27.78 ± 1.60	25.50 ± 1.35
Albumin (g/L)	28.69 ± 0.37	29.07 ± 0.73
Cystatin C (pg/ml)	295.6 ± 28.0	278.4 ± 15.0
Sodium (mmol/L)	141.8 ± 0.83	142.5 ± 0.78
Potassium (mmol/L)	3.33 ± 0.14	3.46 ± 0.15
Calcium (mmol/L)	0.34 ± 0.03	0.31 ± 0.01
Lactate (mmol/L)	1.72 ± 0.25	1.23 ± 0.14

Measures of metabolites and electrolytes in plasma in male offspring of Control and HFD fed fathers at 27 weeks of age. Data are presented as mean ± SEM; *n* = 8 representing one offspring per F0. Statistical analyses were performed using Student’s *t*-test.

**Table 3:** Renal gene expression of offspring at 27 weeks of age.

Paternal Diet	Control (*n* = 8)	HFD (*n* = 8)
Cd36	0.65 ± 0.15	0.57 ± 0.11
Ldlr	0.87 ± 0.06	0.88 ± 0.09
Srebp1	0.94 ± 0.05	0.90 ± 0.04
Srebp2	0.90 ± 0.08	0.85 ± 0.06
Acaca	1.05 ± 0.21	0.86 ± 0.12
Fasn	1.24 ± 0.14	1.10 ± 0.15
Acat 1	0.81 ± 0.02	0.95 ± 0.06 *
Lpl	1.01 ± 0.17	0.87 ± 0.07
Hadh	0.95 ± 0.13	0.82 ± 0.03
Pgc1α	0.99 ± 0.08	0.91 ± 0.03
Pparg	1.10 ± 0.15	1.16 ± 0.05
Cpt1a	1.03 ± 0.12	0.83 ± 0.03
IL-6	1.52 ± 0.21	1.88 ± 0.49
Tnf-α	0.82 ± 0.13	0.90 ± 0.13
Mcp1a	1.18 ± 0.09	0.92 ± 0.11
Tgf β1	1.08 ± 0.06	1.01 ± 0.09
Kim 1	1.32 ± 0.45	0.92 ± 0.15
Ngal	0.98 ± 0.11	0.96 ± 0.09

Data are presented as mean ± SEM (Arbitrary unit); *n* = 8 representing one offspring per F0. Statistical analyses were performed using Student’s *t*-test (* *p* < 0.05 compared to Control rats).

**Table 4:** Histopathological analysis of offspring kidneys at 27 weeks of age.

(%)	Cells Sloughing in Lumen	Absence/Rupture Brush Border	Apoptotic Cells	Debris
Control Father	38.72 ± 2.46	22.77 ± 2.16	0.85 ± 0.40	12.02 ± 0.61
HFD Father	46.86 ± 2.30 *	27.03 ± 1.72 *	1.71 ± 0.74	13.81 ± 1.15

Histopathological analysis of offspring kidneys at 27 weeks of age showing the percentage of cell sloughing, absence of brush border, presence of apoptotic cells and debris in renal tubules. 30 non-overlapping views (200×) from each kidney (*n* = 5 per group) were analyzed by counting the number of tubules showing cells inside lumen, absent or ruptured brush border, presence of apoptotic cells and debris inside the lumen. Data are presented as mean ± SEM. Statistical analyses were performed using Student’s *t*-test (* *p* < 0.05 compared to Control rats).

## Abbreviations

The following abbreviations are used in this manuscript:

Acat 1	Acetyl-CoA acetyltransferase 1
Acaca	Acetyl-CoA carboxylase alpha
Cd36	Cd36 molecule
CKD	Chronic kidney disease
Cpt1a	Carnitine palmitoyltransferase 1A
Ep	Epididymal
Fasn	Fatty acid synthase
GFR	Glomerular filtration rate
Hadh	Hydroxyacyl-CoA dehydrogenase
HFD	High fat diet
Hprt 1	Hypoxanthine phosphoribosyltransferase 1
IL-6	Interleukin 6
Kim1	Kidney injury molecule 1
Ldlr	Low density lipoprotein receptor
Lpl	Lipoprotein lipase
Mcp1	Monocyte chemoattractant protein-1
NEFA	Non-esterified fatty acid
Ngal	Neutrophil gelatinase-associated lipocalin
PAS	Periodic acid-Schiff
Pgc1α	Peroxisome proliferator-activated receptor gamma, coactivator 1 alpha
Pparg	Peroxisome proliferator-activated receptor gamma
q-PCR	Quantitative polymerase chain reaction
Rp	Retroperitoneal
SD	Sprague Dawley
Srebp	Sterol regulatory element binding protein
Tgf β1	Transforming growth factor beta 1
Tnf-α	Tumor necrosis factor alpha
WAT	White adipose tissue
